# 

**Published:** 1890-01

**Authors:** 


					﻿CONTENTS:	PAGB
ORIGINAL ARTICLES.—
Odon tomes. By J. Bland Sutton, F.R.C.S.................. I
Army Veterinary Service. By R. S. Huidekoper, M.D., Vet..12
Progress in Avian Anatomy for the Years 188&-1889. By R. W. Shufeldt,
MD., C.M.Z.S............................................22
Six Cases of Tumors. By Daniel D. Lee, M.D.V.............26
Notes on the Breeding of a Hippopotamus. By W. A. Conklin,
PhD., D.V.S............................................30
Age of the Horse, Ox, Dog, and other Domestic Animals. By R. S. Huide-
koper, M.D., Veterinarian................................32
The Recent Review of Swine-Disease Literature............41
EDITORIAL DEPARTMENT.—
The Journal of Comparative Medicine and Veterinary Archives .... 57
Dr. Huidekoper withdraws from the Veterinary Department of the Uni-
versity of Pennsylvania.................................59
CASES, SELECTIONS, TRANSLATIONS.—
Central Park Menagerie—Autopsy of Kangaroo...............64
Vertiginous Symptoms Consecutive to Clipping in the Horse. By Dr.
Guibert, V S.......................... ..............65
Haematoma—Recurrent Tumor in the Ox. By Dr. Nariman......67
Haematoma in a Cow. By W. H. Ridge, V.M.D...E............68
Experiments upon the Superior Laryngeal Nerve. By L. Breisacher,
V.M.D............................................... 69
SOCIETY PROCEEDINGS..........................................................71
VETERINARY COLLEGE NOTES.....................................................83
NECROLOGY....................................................................84
REVIEW DEPARTMENT........................................................... 86
BOOKS AND PAMPHLETS RECEIVED.................................................88
				

